# Unraveling Proto-Oncogene (ErbB2) Expression in Patients With Carcinoma Head of Pancreas and Chronic Pancreatitis Patients: A Case-Control Study

**DOI:** 10.7759/cureus.54859

**Published:** 2024-02-25

**Authors:** Abhina Mohanan, Pottakkat Biju, Balasubramaniyan V, Gladwin V

**Affiliations:** 1 Surgical Gastroenterology, Jawaharlal Institute of Postgraduate Medical Education & Research, Puducherry, IND; 2 Biochemistry, Jawaharlal Institute of Postgraduate Medical Education & Research, Puducherry, IND; 3 Anatomy, Jawaharlal Institute of Postgraduate Medical Education & Research, Puducherry, IND

**Keywords:** pro-inflammatory genes, pancreatoduodenectomy, real-time polymerase chain reaction, pancreatic oncogenesis and inflammation, pancreatic head enlargement, exocrine pancreas, erbb2/her2/neu, chronic pancreatitis, carcinoma pancreas

## Abstract

Background

The pre-malignant tendency of the normal, non-affected portion of the pancreas is not as well explored as the multicentricity documented in pancreatic cancer cases. In order to ascertain the expression of inflammatory markers and Erythroblastic Oncogene B (*ErbB2*) in the non-affected pancreas in patients with pancreatic cancer, a case-control study was carried out.

Materials and methods

In patients who underwent pancreatoduodenectomy for pancreatic cancer (PC), pro-inflammatory genes and a tumor marker, erythroblastic oncogene 2 (*ErbB2*) in the epidermal growth factor receptor family were analyzed in the pancreatic tissue at the cut surface of the normal pancreas using qRT-PCR. Twenty patients diagnosed with Chronic pancreatitis (CP) after Frey's surgical procedure were selected, and their pancreatic tissues were analyzed as controls. The HPLC-purified primers were designed using National Center for Biotechnology Information (NCBI) software. The primer's specificity was verified for gene expression analysis using the Basic Local Alignment Search Tool (BLAST). The genes under study were normalized using β-actin as the housekeeping gene, and the 2^-ddct^ method was used to compute the fold change compared to the control sample.

Results

Patients with margin-positive were not included. Pro-inflammatory genes (*TNF-α, NF-kβ, *and* COX-2*) had significantly lower foldchange in PC patients compared to the CP group. The CP control group had higher levels of *IL-6* gene expression than the PC group. Patients with pancreatic cancer had a considerably higher expression of the *ErbB2* gene than patients with CP.

Conclusion

The upregulated *ErbB2* gene in the unaffected pancreatic tissue of pancreatic cancer patients, when compared to controls, indicates that the remaining pancreas may have the capacity to cause cancer. Proto-oncogene may play a role in the pathophysiologic process in patients with pancreatic cancer.

## Introduction

The human pancreas is a solid leaf-shaped retroperitoneal organ lying between the first and second lumbar vertebrae in the abdominal cavity; it weighs around 110g and is around 15 cm in length, with well-defined outer borders surrounded by a fibrous capsule covered by connective tissues (CT) [1-4]. The pancreatic parenchyma divides into lobes and lobules. The pancreas is divided into 4 parts: head, uncinate process, body, and tail. The body is situated in the transverse peritoneal space, and the tail extends towards the hilum of the spleen [5]. Functionally, pancreatic parenchyma consists of two main components: exocrine and endocrine pancreas. The exocrine pancreas (acinar cells) occupies more than 95% of the total pancreatic volume (TPV), and the remaining is constituted by thousands of endocrine cells (islets) [5]. 

Structural and physiological abnormalities of both exocrine and endocrine pancreatic tissue result in various diseases. Diagnosing and treating pancreatic disorders might be difficult due to their complexity. From 1990 to 2017, prevalence rates of pancreatitis increased from thirty lakhs to sixty lakhs, with a 13.3 percent rise globally [6]. East Asia, South Asia, and Western Europe had the highest cases [6]. According to a meta-analysis study, the global incidence of acute pancreatitis (AP) was 33.74, and those of chronic pancreatitis (CP) were 9.62 per 100,000 population per year [7]. The mortality in patients with AP was 1.60/100,000, and mortality in patients with CP was 0.09/100,000. In 2019, it was estimated that the global incidence, disability-adjusted life years, and pancreatitis patient mortality were 34.8/100,000 and 1.4/100,000, respectively [7]. Several studies have demonstrated that AP patients may eventually develop chronic symptoms [8-10]. Due to the inflammatory process in the pancreas, the pancreatic cells are lost in CP. Malnutrition and diabetes are linked to a higher likelihood of developing pancreatic cancer (PC) [11]. AP and CP are more commonly seen in males.

The incidence of PC is 5.5 and 4.0 out of every 100,000 for males and females, respectively, worldwide. Less than 10% of patients with PC survive for five years [12,13]. Adjuvant chemotherapy adds a minor survival benefit, but surgery is the only possible curative treatment [14,15]. Approximately 85% of pancreatic tumors are ductal adenocarcinomas, the most prevalent histologic subtype. Relapse following pancreatectomy is common and significantly affects the prognosis. Twenty to 25 percent of PCs are found in the body or tail, but the great majority involve the head region [14,15]. There are no standard targeted marker-based treatments for pancreatic cancer, in contrast to other cancer types, even after significant research efforts [16].

Several genes regulate cell cycle by repairing DNA damage, axon guidance, and chromatin remodeling. Some examples of genes in pancreatic cancer genetics are *KRAS, BRAF, ErbB, TP53, and SMAD4* [16]. We aimed to study the expression of the inflammatory genes and the Erythroblastic- B (a proto-oncogene called *ErbB2* gene in the epidermal growth factor receptor family) by analyzing the pancreatic tissue (n=30) at the cut surface of the normal pancreas from patients who underwent pancreatoduodenectomy for PC. Twenty patients diagnosed with CP after Frey's surgical procedure were selected, and their pancreatic tissues were analyzed as controls for comparison. The *ErbB2* gene was selected in this preliminary study as it is involved in inflammation and cancer. Also, NF-κB/miR-488/ERBB2 axis is reported to modulate the pancreatic tumor growth [17]. Hence, a case-control study was conducted to understand the pathophysiologic processes of CP that lead to pancreatic cancer. The pro-inflammatory genes in the inflammatory cascade studied were the nuclear factor kappa B (NF-kβ), interleukin-6 (IL-6), tumor necrosis factor-alpha (*TNF- α*), cyclooxygenase-2 (*COX-2*) and oncogene *ErbB2* expression in the non-affected pancreas from patients with PC and CP patients as controls were subjected to comparative gene expression analysis by a case-control study.

This article was previously presented as a meeting abstract at the annual conference of the Indian Society of Gastroenterology (ISGCON 2023) on December 24, 2023, and the abstract was published in the Indian Journal of Gastroenterology (IJG).

## Materials and methods

Patients and controls

**Figure 1:**
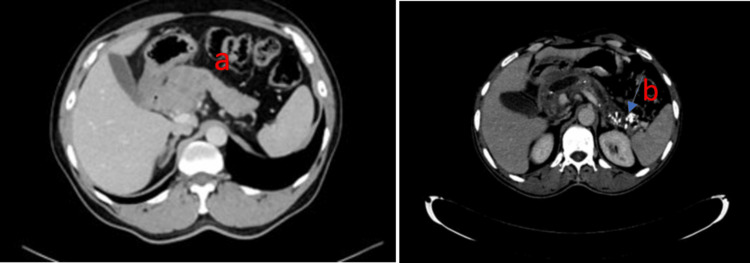
Contrast-enhanced computed tomography (CECT) of the abdomen A- PC patients: a) Carcinoma head of pancreas; B- CP controls: b) Area of CP patients calcification and pancreatic ductal dilatation and pancreatic atrophy PC: Pancreatic cancer; CP: Chronic pancreatitis

This study was conducted from January 2018 to December 2021 in the Department of Surgical Gastroenterology, JIPMER. After getting approval from the Institutional Ethical Committee (IEC) for human studies, written informed consent was obtained from all the patients and control subjects. In patients with suspicion of the diagnosis, a biopsy or cytology was performed. Based on the clinical presentation of weight loss and occasional epigastric discomfort, with or without exocrine and endocrine insufficiency, CP was diagnosed. In order to verify the diagnoses, assess the pancreatic features, and rule out complications connected to the condition, each patient underwent a contrast-enhanced computed tomography (CECT) scan of the abdomen (Figure 1).

**Figure 2:**
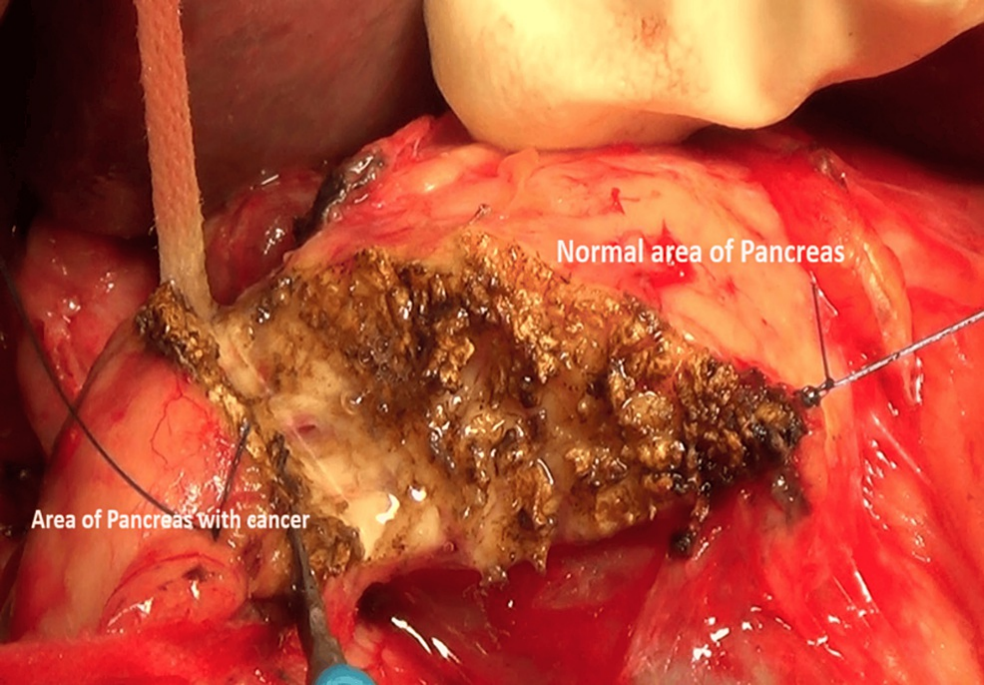
Tissue specimen of normal, unaffected part from PC patients PC: Pancreatic cancer

Thirty PC patients between 18-70 years participated in the study. Quantitative real-time polymerase chain reaction (qRT-PCR) analysis was performed on pancreatic tissue (a normal part of the pancreas at the cut surface margin of the neck) collected from PC patients who underwent pancreatoduodenectomy (Figure 2). Twenty patients who underwent head resection/head coring (Frey's surgical procedure) for CP had their pancreatic tissues evaluated as controls. Patients with CP were excluded in the cases, and those with carcinoma were excluded from the controls. Exclusion criteria for the study participants were patients with acute pancreatitis, a history of any pancreatic surgery or major gastrointestinal surgery (gastrectomy, colectomy), patients undergoing antioxidant therapy, active or uncontrolled infection, and other malignant conditions, patients on other chronic, inflammatory disease treatment, and psychiatric or neurologic problems, pregnancy or lactation. The sample size was determined using OpenEpi software, version 3.1, under the assumptions of 95% CI and 80% power. Relevant demographic details, clinical symptoms, comorbidities, and imaging characteristics were documented. Systematic examination of weight and height were assessed, and BMI was computed.

Gene expression study

**Figure 3:**
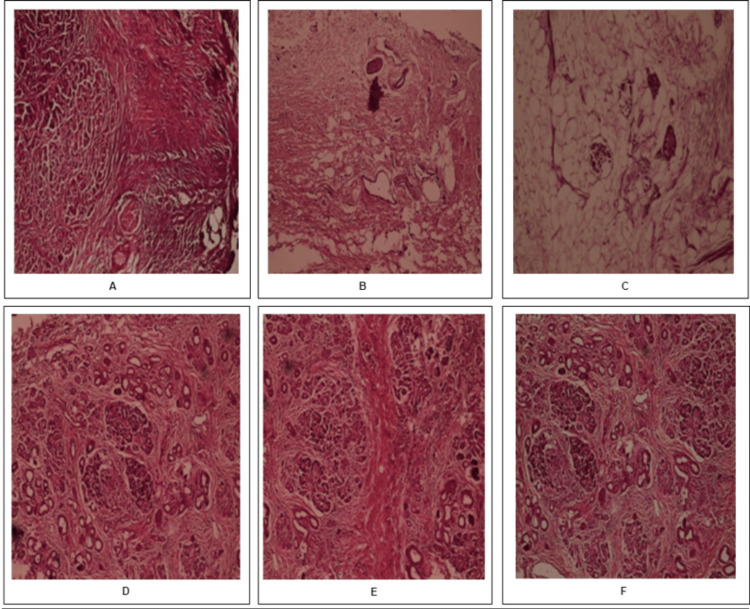
H and E-stained sections of the pancreas from CP controls 1. Sections from the pancreas with dilated ducts, diffused fibrosis with residual Islets of Langerhans, complete loss of acinar cells, and marked lipomatous atrophy (A, B, and C-100X) 2. Sections from the pancreas showing cellular atrophy and calcification (D, E, and F-100X) CP: Chronic pancreatitis

**Figure 4:**
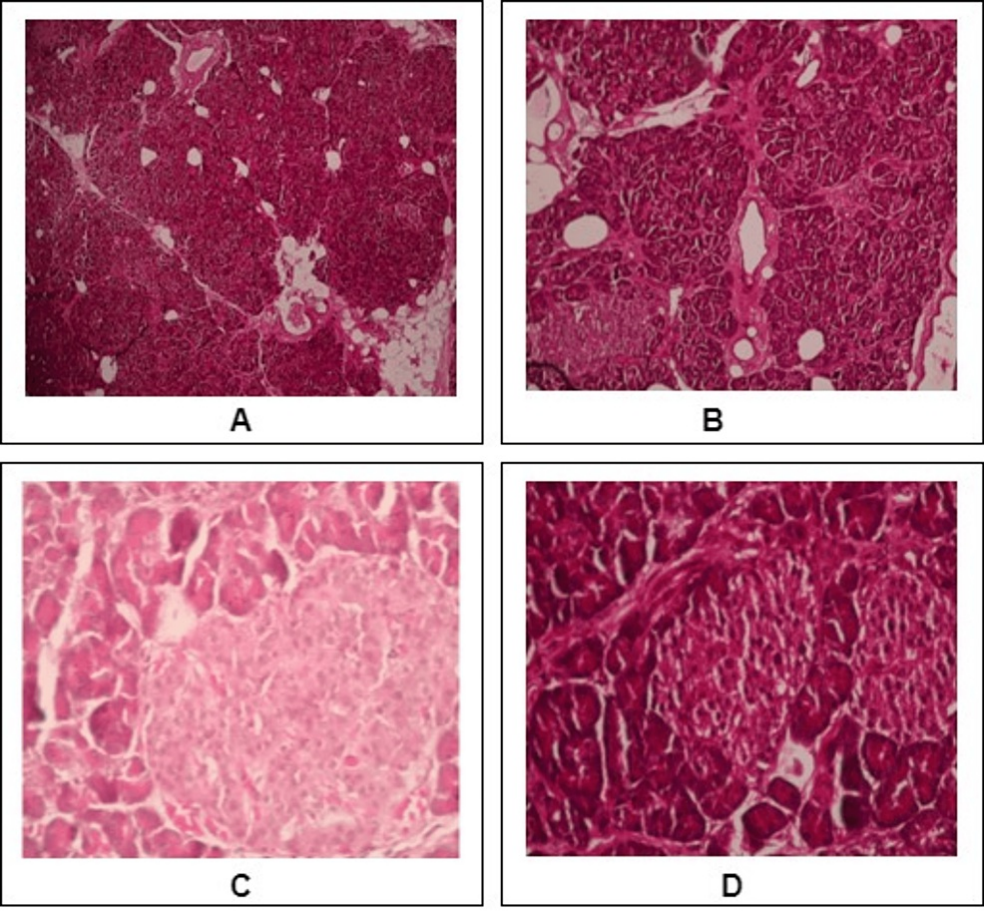
H and E-stained sections of the normal part of the pancreas from PC patients (A, B-100X; C, D-400X) c. Normal Islets of Langerhans and Acini

Histopathological examination was done for all specimens collected from the operation theatre. For histological analysis, the pancreatic tissue, fixed in 10% neutral buffered formalin (NBF), was embedded in paraffin. Using the hematoxylin and eosin (H&E) staining method, the tissue sections of 5mm (Figures 3-4) were used to verify and confirm the normal pancreas area in PC and CP before further analysis of gene expression study.

The remaining pancreatic specimens were washed in chilled PBS under aseptic conditions and kept at -80˚C in RNA later solution to extract total RNA. The RNA was isolated using the (Qiagen) RNeasy mini kit method. After measuring the purity and concentration of the total RNA by A260/A280 and A260/A230 ratios using a nanodrop spectrophotometer, the collected RNA was stored at -80˚C. Using the Primescript RT reagent kit, isolated RNA was converted to cDNA. The following were the PCR conditions for cDNA amplification: Initial denaturation was done at 95˚C for 5 min, 35 cycles of denaturation, annealing, and extension (at 95˚C for 45s, 64˚C for 45s, and 72˚C for 1 min, respectively) and the amplified converted cDNA was stored at -20˚C. HPLC-purified primers (Eurofins) specific to the genes of interest (Table 1) were utilized for gene expression analysis. National Center for Biotechnology Information (NCBI) software-designed HPLC-purified primers were synthesized. The Basic Local Alignment Search Tool (BLAST) verified the specificity of the primer for gene expression analysis. Mastermix was prepared using TB Green Premix Ex Taq II (Takara Bio Inc, India), and Real-time polymerase chain reaction (RT-PCR) with Quant 5 studio Applied Biosystems (ABI) was carried out. The list of qPCR reaction mixture reagents and thermal conditions for real-time-PCR (Tables 2-3). The expression of each target gene in tissues from CP patients was compared with that of normal pancreatic tissue in PC after being normalized to the housekeeping gene (β-actin). The β-actin was the housekeeping gene used to normalize the genes, and the 2^-ddct^ formula was used to compute the fold change relative to the control sample using the method prescribed [18].

**Table 1:** HPLC-purified primers description β- actin: Beta actin; TNF-α: Tumor-necrosis factor-alpha; NF-κB: Nuclear factor kappa B; COX-2: Cyclooxygenase-2; IL-6: Interleukin-6; ErbB2: Erythroblastic oncogene-B

Sl. No.	Gene name	Primer sequence (5"- 3")	Product Size (bp)	Accession No.
1.	β-actin	AGCTCGCCATGGATGATGA TGCCGGAGCCGTTGT	54	DQ 661647
2.	NF-κB	GCGGGAGAGGGGATTCCCTGCGGCCCCG CGGGGCCGCAGGGAATCCCCTCTCCCGC	364	NM 001077494.3
3.	TNF-α	ATCTGGAGGAAGCGGTAGTG AATAGGTTTTGAGGGGCATG	103	NM 182703.6
4.	IL-6	CATCCTCGACGGCATCTCAG ACCAGGCAAGTCTCCTCATTG	162	XM 005249745.6
5.	COX-2	GTTCCACCCGCAGTACAGAA AGGGCTTCAGCATAAAGCGT	106	NM 000963.4
6.	ErbB2	AGCTGGCGGCCTTGTG CCGGTGCACACTTGGGT	79	NM 004448.4

**Table 2:** List of reagents of the qPCR reaction mixture

Reagent	Volume (μL)
TB Green Premix Ex Taq II (Tli RNaseH Plus) (2X)	10 μl
PCR Forward Primer (10 μM)	0.8 μl
PCR Reverse Primer (10 μM)	0.8 μl
ROX Reference Dye (50X)	0.4 μl
Template (cDNA)	2 μl
Sterile purified water	6 μl
Total	20 μl

**Table 3:** Thermal conditions of real-time PCR

Steps	Thermal profile
Initial Denaturation	95℃ for 30s (1 cycle)
PCR	95℃ for 5s (40 cycles)
Annealing	62℃ for 34s (40 cycles)
Dissociation Stage	72℃ for 30s (1 cycle)

Statistical analysis

All the statistical analyses were performed using the software Statistical Package of Social Service (SPSS) version 21.0. The mean ± standard deviation or median is used to express the results (IQR). Where appropriate, the Mann-Whitney (non-parametric variable), independent t-test (parametric variables), or Pearson chi-square tests were used to ascertain the differences between the groups. A p-value of less than 0.05 was used to establish the significance level at a 90% confidence level.

## Results

The demographic, clinical, and laboratory features of the PC patients and controls are given in Table 4. The gender, BMI (Kg/m^2^), history of alcohol intake, smoking, incidence of diabetes mellitus, and diet of each group were not different. In our study group, combined alcohol and smoking addiction were noted in 10 patients (33.3%), and none of the women were alcoholics. Diabetes mellitus was reported in 11 (36.6%) PC patients, and nine (45%) CP control participants had diabetes. Steatorrhea, indicating exocrine insufficiency, was observed in just one (3.3%) of PC patients and one (5.0%) of CP controls. The biochemical parameters of PC and CP patients are given in Table 5, respectively. Pancreatoduodenectomy was performed in 30 patients with PC. Thirteen patients with CP underwent head coring Frey's procedure, and seven underwent pancreatoduodenectomy. Mild to extensive fibrosis was observed in six (20%) of patients with PC, whereas the same was observed in 19 (95%) in CP controls.

**Table 4:** Demographic and clinical symptoms of patients and controls All parameters – chi-square tests, BMI- Body mass Index; $ Mean±SD, S.D- standard deviation

Sl.No.	Symptoms	PC Patients, n (%)	CP controls	p-value
1.	Age (years)^$^	51.43±11.13	40.9±11.36	0.002
2.	BMI (kg/m^2^)^ $^	21.62±5.31	20.28±3.59	0.332
3.	Gender, n (%)			0.064
	Male	13 (48.1)	14 (51.9)	
	Female	17 (73.9)	6 (26.1)	
5.	Diabetes	11 (50)	9 (45)	0.556
6.	Alcohol drinkers	10 (50)	10(50)	0.239
7.	Smokers	11 (50)	9 (45)	0.556
8.	Abdominal pain on admission	8 (26.0)	20 (100)	<0.001
9.	Jaundice	20 (66.7)	0 (0)	0.00
10.	Steatorrhea	1 (3.3)	1 (5.0)	1.00
11.	Dyspeptic symptoms	3 (10)	1 (5.0)	0.641
12.	Melena	6 (20.0)	1 (5.0)	0.219
13.	Vomiting	11(36.7)	8 (40)	0.842
14.	Pruritis	15 (50)	0 (0.0)	<0.001
15.	Loss of weight	19 (63.3)	8 (40)	0.105
16.	Loss of appetite	14 (46.7)	12 (60.0)	0.355

**Table 5:** Biochemical parameters in patients and controls All parameters- Independent t-test except total bilirubin, AST, ALT, ALP, GGT, urea, creatinine, amylase, CA199-Mann Whitney test; RBS- Random Blood Sugar, AST- Aspartate aminotransaminase, ALT- Alanine aminotransaminase, ALP- Alkaline Phosphatase, GGT- Gamma-glutamyl transferase in LFT- Liver Function Test, Hb-Hemoglobin, TLC- Total Leucocyte Count; mg/dl-milligram per decilitre, g/dl- gram per decilitre, IU/L-International unit per liter ul-microlitre mEq/L-milli equivalent per liter

Sl. No.	Biochemical parameter	PC patients (Mean± S.D)	CP Control (Mean± S.D)	p-value
1.	Hb (g/dl)	10.96 ±1.81	11.58±2.5	0.317
2.	Platelets (*10^3^ ul)	2.99 ±1.02	2.7 ±0.86	0.365
3.	TLC (ul)	20705.3 ±61052.13	10151.05 ±3896.16	0.445
4.	Glucose (RBS) (mg/dl)	129.72 ± 84.32	109.15 ±35.43	0.87
5.	Total bilirubin (mg/dl)	4.37± 5.31	0.759 ±0.462	0.003
6.	Total protein (g/dl)	6.7 ±0.55	6.9 ±0.86	0.382
7.	Albumin (g/dl)	3.40±0.521	3.86 ±0.82	0.07
8.	Globulin (g/dl)	3.31 ±0.45	3.07 ±0.34	0.143
9.	AST (IU/L)	60.39 ±38.81	37.9 ±33.28	0.017
10.	ALT (IU/L)	56.61 ±50.38	36.0 ± 28.54	0.061
11.	ALP (IU/L)	336.28±242.86	223.85 ±294.94	0.007
12.	GGT (IU/L)	191.28 ±207.46	80.8 ±55.98	0.052
13.	Urea (mg/dl)	27.9 ±30.92	25.2 ±25.84	0.54
14.	Creatinine (mg/dl)	1.04 ±1.20	1.26 ±1.42	0.208
15.	Sodium (mEq/L)	133.3 ±4.3	133.35 ±4.7	0.969
16.	Potassium (mEq/L)	4.20 ±0.73	4.24 ±0.63	0.849
17.	Amylase (U/L)	259.02±340.35	169.33±246.66	0.23
18.	CA19-9 (U/mL)	1157.53±3861.90	16.36±6.03	0.42

**Figure 5:**
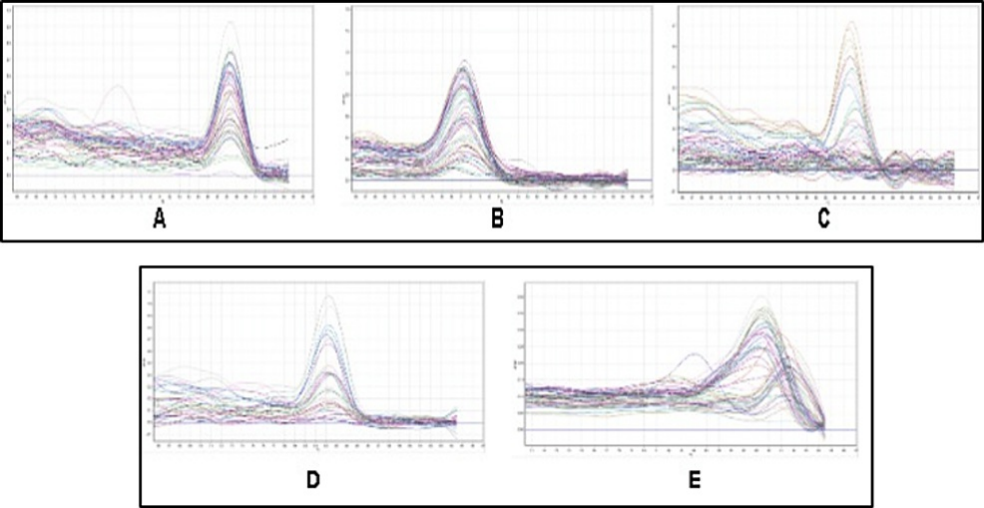
Melt curve analysis of the genes under study (A. TNF- α; B. NF-kβ; C. IL-6; D. COX-2; E. ErbB2) *TNF-α*: tumor-necrosis factor-alpha, *NF-κB*: nuclear factor kappa B, *COX-2*: cyclooxygenase-2, *IL-6*: interleukin-6, *ErbB2*: Erythroblastic Oncogene B

To explore the molecular mechanisms of inflammation in patients and control groups, we studied the gene expression of pro-inflammatory genes in the inflammatory cascade, *TNF- α, NF-kβ, IL-6, and COX-2*. The gene expression analysis is given in Table 6. Melt curve analysis of the genes is given in Figure 5. In the present study, the median fold change of *TNF-α, NF-kβ, and COX-2* was significantly lower in PC patients than in the CP group. Compared to the PC group, CP patients had higher IL-6 gene expression levels. In PC patients, the median fold change of *TNF-α, NF-kB, and COX-2* was significantly lower than that of CP controls. Tissues in PC patients and controls were subjected to proto-oncogene *ErbB2* gene expression analysis to explore the pathophysiologic processes of PC thoroughly. Our findings observed that *ErbB2* is overexpressed in PC patients compared to control CP tissues (Table 6). The *ErbB2* gene was significantly upregulated in PC patients compared to CP controls.

**Table 6:** Gene (mRNA) expression analysis in the pancreatic tissue of patients and controls *TNF-α*: tumor-necrosis factor-alpha, *NF-κB*: nuclear factor kappa B, *COX-2*: cyclooxygenase-2, *IL-6*: interleukin-6, *ErbB2*: Erythroblastic Oncogene B

Sl. No.	Fold change	PC case	CP Controls	p-value
		Median (IQR)	Median (IQR)	
1.	TNF-α	1.40 (0.45-4.89)	4.5 (1.48- 7.75)	0.003
2.	NF-kβ	0.88 (0.50- 2.33)	2.3(1.41- 6.89)	0.001
3.	IL-6	1.33 (0 .40 -4.13)	3.3 (0.52-4.89)	0.11
4.	COX-2	0.86 (0.25-4.77)	3.56 (1.68- 8.13)	0.02
5.	ErbB2	0.72 (0.41-1.88)	0.64 (0.20- 0.75)	0.006

## Discussion

More than 60% of PC patients had jaundice and weight loss. Patients with head tumors frequently had jaundice, while body/tail cancers usually showed indications of pain and weight loss [15]. Because pancreatic head tumors obstruct the common bile duct, they typically result in developing jaundice and subsequent hyperbilirubinemia. In CP controls of our study, 95% of the participants experienced upper abdominal pain limited to the upper right or left quadrant. Other symptoms included appetite loss and loss of weight, followed by jaundice and vomiting, which is consistent with other research [19]. The pain is usually deep due to the retroperitoneal location of the pancreas. Chronic alcohol misuse is frequently linked to CP, which is accompanied by loss of acinar (exocrine) and islet (endocrine) cells, as well as inflammatory changes with cellular fibrosis [15]. When alternative pain management methods proved ineffective, they also underwent surgery, and the tissue specimens were evaluated for gene expression.

The median fold change of *NFk-B, TNF-α, and COX-2* genes in the non-cancerous part of PC tissue patients was significantly lower than CP controls in our study. *TNF-α, IL-6, and COX2* are a few of the many inflammatory genes that NF-kβ regulates. Pancreatic acinar cells release *TNF-α* [20]. *TNF-α* has also been linked to the development of pancreatitis by promoting early inflammatory responses [20]. According to their research, the rat pancreas had peak levels of *TNF-α, IL-6*, and its receptors gene expression at different times. These results suggest that (i) TNF-α may be associated with the onset of CP and (ii) IL-6 may contribute to inflammation [20]. TNF-α is a mitogenic cytokine and IL-6 fibrogenic cytokine [21]. Vascular endothelial and infiltrating cells were the primary sources of IL-6 [20]. Although there may not be a direct link between TNF-α in developing pancreatic fibrosis, TNF-α may play a role by activating the pancreatic fibrosis-promoting enzyme TGF-β.

Pro-inflammatory cytokines, such as TNF-α and IL-6, are expressed more actively due to metabolic inflammation and oxidative stress. To slow the onset and advancement of the disease, metabolic and vascular function, and oxidative inflammation cascades may be modified [22]. Discovering novel treatments to lower the likelihood of recurrent AP progressing to CP and then to carcinoma changes may be possible by measuring DNA damage and inflammatory markers and determining the clinical scenario from the time of AP development. *TNF-α* and its receptors are crucial players in the emergence of acinar cell death in this paradigm because TNF-α triggers apoptosis, and NF- kB gets activated. Decreased mRNA levels of NF-κB indicate that the NF-κB signaling pathway has been less activated in PC patients than in CP controls. Numerous factors like infections, pro-inflammatory cytokines, and cellular stress may contribute. *NF-kB*, which was first discovered to be one of the inflammatory nuclear factors, is a transcription factor often found as a dimer in most cells and helps identify how signaling processes affect physiology and gene expression [23]. This crucial transcription factor is involved in macrophage activation and is essential in initiating inflammatory reactions [24]. A study in CP condition reported that up-regulation of *COX-2* expression in inflammatory conditions (in CP controls) develops clinical signs and symptoms of inflammation-related diseases [25].

A study in WBN/Kob rats proposed that distinct chronic pancreatitis can be identified even at 24 weeks, and pancreatic histology is not entirely reversible [20]. Observed parenchyma degradation and severe fibrosis in the earlier stages of inflammation [20] and decreased fibrosis at 20 and 24 weeks could have been caused by the regeneration of acinar cells [20]. The histologic alterations of inflammation occurred at 12 weeks [20] when IL-6 and its receptors' mRNA expressions spiked. IL-6 may contribute to both inflammation and pancreatic fibrosis. IL-6-producing cells found in the vascular wall were responsible for the IL-6 mRNA expression in the pancreas of the rat model at 4 and 8 weeks. In our study also, IL-6 was downregulated in PC patients compared to CP control tissues. Our patients had undergone surgery in their later stage of pancreatitis when they could not manage by other means of treatment. These investigators suggested that IL-6 and TNF-α have a role in developing pancreatitis. Our study conducted in human samples also identified the role of IL-6 and TNF-α in developing pancreatitis. In CP controls, edema, Calcification, and fibrosis (mild, moderate to marked) were noticed at this stage. The abnormalities that first manifested in the pancreas at 12 weeks were inflammatory cell infiltration, edema, and fibrosis.

The expression of *ErbB2* in patients with PC was upregulated compared to CP control tissues. Patients with pancreatic cancer and chronic pancreatitis often have overexpressed tyrosine kinase receptors, like *ErbB2* of the epidermal growth factor receptor family [24]. Though *ErbB2* is involved in pancreatic inflammation and oncogenesis [24], NF-κB/miR-488/ErbB2 axis is reported to modulate pancreatic tumor growth [17]. *ErbB2* is involved in PC also. Because of delayed diagnosis and ineffective treatment options, the prognosis for pancreatic cancer is among the worst of any cancer [17]. The pancreatic cancer cell cycle, viability, and apoptosis of the disease are all impacted by NF-κB transcriptionally inhibiting *ErbB2* expression, as demonstrated by data from Chinese patients with PC [17]. The researchers developed transgenic mice lines that overexpress human *ErbB2* to clarify the function of the oncogene in the exocrine pancreas [24].

While early pancreatic lesions have been demonstrated to upregulate *ErbB2*, transgenic mice developed substantial lymphocytic infiltrations and focal regions of chronic inflammation instead of tumors or preneoplastic lesions. The pancreas of transgenic mice with proliferated acinar and ductal cells did not follow overexpression of *ErbB2*. This mouse model may shed light on the function of *ErbB2* in pancreatic inflammatory processes [24]. People with CP, a recognized risk factor for PC in humans, had enlarged pancreatic heads due to acinar cells overexpressing *ErbB2* [26]. According to their findings, upregulated ErbB2 in pancreatic acinar cells induces inflammatory changes rather than a tumor, indicating a potential role for *ErbB2* in the pancreatic chronic inflammatory processes [24]. However, our study observed upregulated *ErbB2* expression in PC tissues rather than chronic inflammatory control tissues, which is in accordance with the study wherein overexpressed *ErbB2* protein was found to have a major role in cell proliferation and differentiation [27]. Pancreatic head enlargement is a recognized risk factor for pancreatic cancer in humans [28]. As the non-cancerous PC tissues were taken from diseased individuals, the gene expression would also have spread to adjacent cells. An increase in *ErbB2* expression in tissue samples from PC patients was noted, particularly in patients with enlarged pancreatic heads [24]. Our PC tissues were taken from patients who had the potential to provide normal pancreatic tissue at operation. The normal pancreatic tissue was removed from the normal part of the pancreas and operated on for pancreatic adenocarcinoma of the pancreas, where there was a significant proportion of normal pancreatic tissue at the cut margin. In an immunohistochemistry analysis study by Friess et al., the *ErbB2* oncogene was increased in regions adjacent to cancer regions. Hence, this could be a reason for the upregulation of the *ErbB2* gene observed in our study [26]. Even with the histological evidence of being a normal part of the specimen from a pancreatic cancer patient, when a patient with adenocarcinoma underwent curative resection and the tumor was fully resected, making sure that the tumor had not metastasized to any nodes, the patient passed away within days from recurrent malignancy [14]. As in many cases where gross examination did not reveal a spread of cancer, this could be possibly explained by pointing out the fact that the adjacent areas of the pancreas may be carcinogenic and may have a role in the oncogenesis process in patients with PC.

It was found that when standard chemotherapy is combined with lapatinib regimens, which simultaneously block ErbB1 and 2 receptors, pancreatic cancer cell growth and proliferation are decreased, and apoptotic cell death is promoted [27]. Recurrence of the disease or metastasis of the disease is common, even in patients receiving early surgical therapy. Gaining further insight into the molecular processes by studying more genes that precede or initiate this deadly illness is necessary to develop methods for identifying preinvasive pancreatic neoplasms and to create more efficient treatment regimens [29].

The strength of this study was that all confirmed cases (diagnosed by histopathologic reports and CT scan) of PC and CP that were unmanageable by medications and endoscopic therapy underwent either Frey's procedure or pancreatoduodenectomy, and demographic characteristics, biochemical parameters, and the mRNA expression of tissue specimens were evaluated. However, this study has some limitations. Due to technical difficulty and high cost, a protein expression could not have been done. These laboratory parameters' cost-effectiveness, easy applicability, and reproducibility remain fundamental problems in daily clinical practice.

## Conclusions

Though the pro-inflammatory genes viz, *NFk-B, TNF-α, and COX-2* genes in non-cancerous part of PC tissue patients were significantly downregulated in normal tissue of PC patients when compared to controls, the patients with pancreatic cancer had higher levels of the Erythroblastic oncogene B, *ErbB2* in their unaffected pancreatic tissue. This suggests that the remaining pancreas may have the capacity to cause cancer and may play a role in the pathophysiologic process of patients with pancreatic cancer.
